# Optimization of an improved, efficient and rapid *in vitro* micropropagation protocol for *Petunia hybrida* Vilm. Cv. “Bravo”

**DOI:** 10.1016/j.sjbs.2021.05.018

**Published:** 2021-05-13

**Authors:** Iqra Farooq, Z.A. Qadri, Z.A. Rather, Imtiyaz T. Nazki, Neelofar Banday, Sadaf Rafiq, Khalid Z. Masoodi, Ahmed Noureldeen, Sheikh Mansoor

**Affiliations:** a Division of Floriculture and Landscape Architecture, SKUAST-Kashmir, Srinagar, J&K 190025, India; b Division of Plant Biotechnology, SKUAST-Kashmir, Srinagar, J&K 190025, India; c Department of Biology, College of Science, Taif University, P.O.Box 11099, Taif 21944, Saudi Arabia; d Division of Plant Biochemistry, SKUAST Jammu, J&K 180009, India

**Keywords:** Callus, In vitro, Micropropagation, Organogenesis, *Petunia hybrida*, Proliferation, Tissue culture

## Abstract

An efficient protocol for *in-vitro* propagation of an important ornamental crop, *Petunia hybrida* Vilm. Cv. “Bravo” was developed. The explants that were used to carry out the experiment were Leaf segments, nodal segments and shoot tips. Nodal segments recorded highest per cent asepsis followed by shoot tips and leaf segments. Asepsis was found to be highest when the explants were sterilized with Fungicide (Carbendazim) 0.02% for the duration of 30 min followed by 0.1% HgCl_2_ for duration of 10 min and then ethanol 70% for 10 s. Longer duration of the sterilant treatment showed more necrotic effects on the explants, thus mercuric chloride treatment when given for 5 min proved to be more effective in terms of survival of the explants. Maximum establishment per cent was recorded in Murashige and Skoog (MS) media fortified with BAP (1.5 mg L^−1^) and IBA (0.5 mg L^−1^) in shoot tips and nodal segments, i.e. 97.90 and 95.74% respectively. Callus was efficiently induced and developed when PGR amalgamation of BAP (0.1 mg L^−1^) and 2,4-D (1.5mg L^−1^) was used. Kinetin at the concentration of 2.0 mg L^−1^ along with IBA at 0.5mg L^−1^ recorded highest callus regeneration in both leaf and internodal segment derived callus. Maximum proliferation percent of shoots (97.90%), highest number of shoots (20.50 explant^−1^) and maximum length of shoot (2.70 cm) was recorded in PGR combination of IBA and BAP both at 0.5 mg L^−^1 concentration level. Rhizogenesis was recorded to be highest in the MS media containing IBA 1.00 mg L^−1^. Best hardening media which recorded maximum survival per cent 92.50% was noticed on the media formulation comprised of equal ratio of perlite and vermiculite mix, under poly house conditions.

## Graphical abstract

**Figure:**
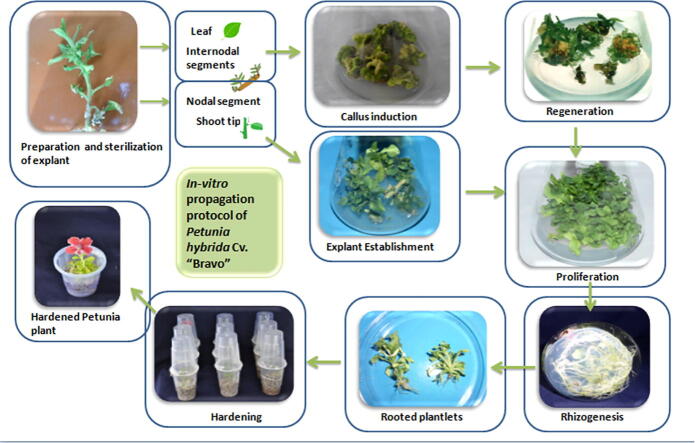

## 1 Introduction

*Petunia hybrida* Vilm. is an annual or perennial plant that belongs to the family solanaceae. Petunias have a great profusion of bloom under all conditions which makes them useful and popular aesthetically as well as commercially. It is a decorative plant, grown for its beautiful flowers in beddings, borders, hanging baskets, window boxes, pots and containers. In warm climates petunias are perennials but are used as annuals in temperate zones (Bailey, 1976, Armitage, 1985). Besides, petunias having a significant importance as ornamental crop, these have also been known as one of the most excellent model crops for studies of gene regulation and genome structure, since the system combines innumerable and excellent technical features with a broad range of research possibilities (Singh, 2014). Hybrid petunia is mainly cultivated through seeds and the vigour and quality can be seen degrading in the further generations because of the segregation that takes place. In order to maintain the F1 progeny for further multiplication and to maintain the vigour of particular cultivar, micropropagation plays an important role. Micropropagation refers to the culture of tissues of the selected plants that are grown in an asceptic condition on a medium containing macro and micro nutrients to produce disease free and true to type plants (Mohammed, 2020, Khan et al., 2020, Arvas et al., 2018). The principle behind the concept of tissue culture is totipotency which refers to the ability of cell to regenerate into a whole plant (Ahmad et al., 2006, Ahmad et al., 2007, Kaya and Huyop, 2020). Apart from this it encourages the chances of semi to complete perennial nature of any specific variety which otherwise is grown as an annual under temperate conditions. Therefore, micropropagation of F1 hybrid petunia delimits the possibility of maintaining quality characteristics, promotes chances of somaclonal variations and aids in high success rate of propagation which is otherwise around 50–60% in F1 hybrids and if aided by priming can enhance only 5–10%. Callus development in petunia has numerous applications like secondary metabolite production, somatic embryogenesis and direct organogenesis for clonal propagation, gene transformation in addition to the studies on cell division, elongation and differentiation (Ozyigit, 2008, Praveen et al., 2010, Kaya et al., 2013). The type of the explant also plays a significant role in *in-vitro* propagation of Petunia (Clapa and Cantor, 2006, Abu et al., 2010, George et al., 2008). The optimization of the plant propagation technology reduces the production costs without compromising the quality (Hamid et al., 2015). Improvement of the plant’s aesthetic parameters, creation of novel variations and micropropagation of ornamental plants are the economic goals for the commercial ornamental industry (Rout and Jain, 2005, Khan et al., 2020). Therefore it is evident that there is a need to establish *in-vitro* propagation protocol of petunia that will not only aid in the maintenance of qualitative and quantitative characters but will create a platform for further elite propagation and breeding programmes viz., cultivar improvement through *in-vitro* mutagenesis and somaclonal variations. Multiplication of Petunia through shoot tips (Dash and Singhsamant, 1990) and through leaf and internodal segments (Rao et al., 1973) has been reported. Abu et al. (2010) has demonstrated the auxin-cytokinin combination effect on shoot proliferation and regeneration of Petunia.

The present study was carried out with the aim of optimization of growth regulator regimes for *in-vitro* propagation of *Petunia hybrida* and *ex-vitro* standardization of hardening of *in-vitro* propagated rooted plantlets.

## 2 Material and methods

### 2.1 Preparation and sterilization of plant material

**Fig. 1:**
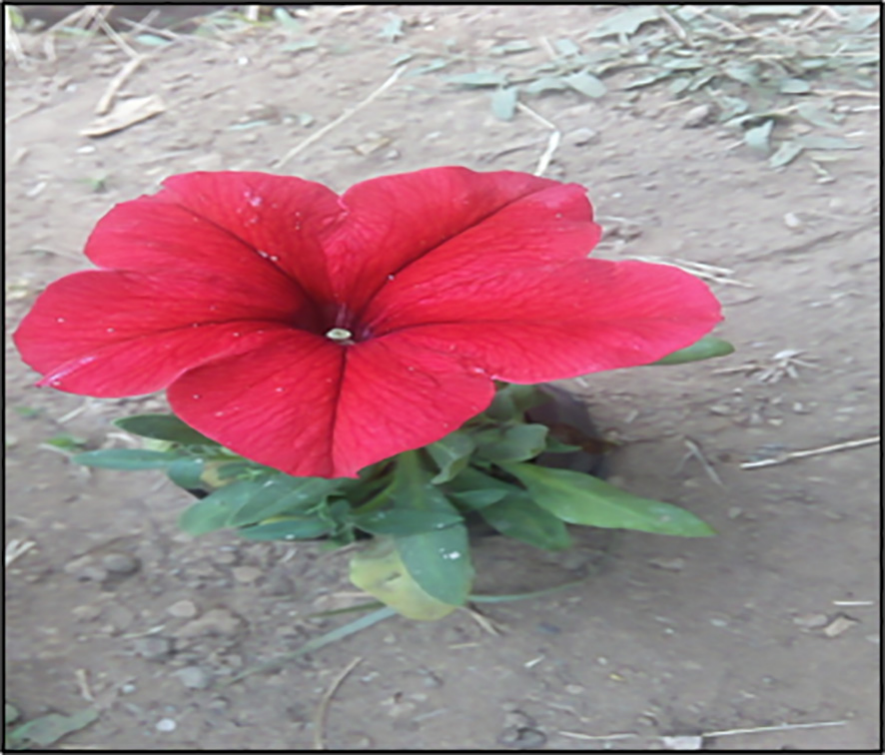
Petunia cultivar (“Bravo”) selected for the investigation.

The study was carried out at Plant Tissue Culture Laboratory of The Division of Floriculture and Landscape Architecture, Sher-e-Kashmir University of Agricultural Sciences and Technology of Kashmir, Shalimar. Actively growing shoots (7–10 cm) of petunia cv. ‘Bravo’ (Fig. 1) were collected from plants grown in polyhouse conditions. The adhering dirt and dust were washed off under running tap water. Younger leaves in shoot tips were retained, as the outer mature leaves were removed with a sharp scalpel. In case of nodal segments, both top and basal re-cuts were given and the explants were reduced to the manageable size of and 1.0, 2.0 and 3.0 cm, and leaf explants were prepared as the discs of 1 cm^2^ size. Later the explants were strenuously quivered in Tween-20 surfactant fortified with different concentrations of the fungicide- Carbendazim for different time durations which were later washed off with tap water followed by washing distilled water. Mercuric chloride (HgCl_2_) at the concentration of 0.1% was then used to treat the explants for different time durations and later ethanol treatment for 10 s was given under the laminar flow hood. Per cent asepsis of the explants was recorded after two and four weeks of culture and per cent survival was recorded after four weeks of culture.

### 2.2 Culture media and culture conditions

The nutrient media employed for the development of propagation protocol of *Petunia hybrida* was MS (Murashige and Skoog, 1962) containing the macro elements, microelements, and vitamins. Sucrose (3%), myo-inositol and Plant Growth Regulators (PGR’s) were put in to the media as per the requirement for each step. Media was maintained at 5.7pH and solidified with agar agar. The test-tubes and flasks containing the prepared culture media were autoclaved for 15–20 min at 121 °C and 15 psi. The cultures were later incubated under controlled conditions in a culture room with 24 ± 1 °C temperature and a 16:8 h, light: dark system. Shoot tip, nodal segment and leaf segment were used as explants for standardization of establishment experiments. Leaf explants failed to regenerate adventitious shoots in exploratory investigations and hence were excluded from the establishment experiments. Explants after the surface sterilization were inoculated on the media carrying different PGR combinations of auxin, IBA (Indole-3-butyric acid) and cytokinin, BAP (6-Benzylamino Purine) for establishment. Callus induction was observed on leaf segments and internodal segments placed on basal MS media consisting of different combinations of BAP, 2,4-D (2,4- dichlorophenoxyacetic acid) and NAA (Naphthalene acetic acid). The percentage of callus induction, callus fresh weight (g explant^−1^) and type of callus were the parameters documented after 4 weeks of incubation. Callus sections having uniform size and age, originating from leaves and internodal segments were placed on regeneration media boosted with various combinations of cytokinins (BAP and Kinetin) and auxins (NAA and IBA). Percentage of the callus developing shoots and shoot number per callus piecewere then observed. Shoot proliferation was noticed as good number of axillary shoots was induced and multiplied *in-vitro* from establishment and callus regeneration cultures. Uniform sized microshoots were divided from the shoot clumps and later inoculated on proliferation media containing different PGR combinations of IBA and BAP. Per cent proliferation, shoot length (cm) and shoot number per explant were observed after 6 weeks of inoculation. Rhizogenesis of microshoots was standardized on rhizogenesis media containing various levels of auxins (IBA and NAA). Uniform lateral microshoots were separated from the proliferated clumps and transferred to the media. Rhizogenesis percentage and roots per shoot were studied during the course.

### 2.3 Acclimatization

Six formulations of different types of growing media viz., sand, perlite and vermiculite were engaged for the standardization of hardening procedure of the rooted plantlets. The components for particular media formulation were mixed in oven dried containers and then put in cloth bags enveloped in aluminum foil sheets, autoclaved for an hour. Rooted plantlets of petunia obtained were shifted to polypropylene containers for hardening purpose under laminar air flow chamber. Another polypropylene glass was inverted over each container containing hardening media and the rims of both containers were sealed with para film strip. These hardening containers kept in the culture chamber with optimum light duration and intensity. After some time, a sign of establishment was observed as new leaves appeared from the survived plantlets. Small holes were made in inverted glass after 10 days of transfer which were later removed. Observations on number of days taken for establishment and *ex-vitro* survival % of rooted plantlets after 4 weeks were recorded during this transition phase.

### 2.4 Statistical analysis

The data compiled during the current study for different parameters was statistically analyzed under completely randomized design (CRD) with four replications.

## 3 Results

### 3.1 Culture asepsis and explant establishment

**Fig. 2:**
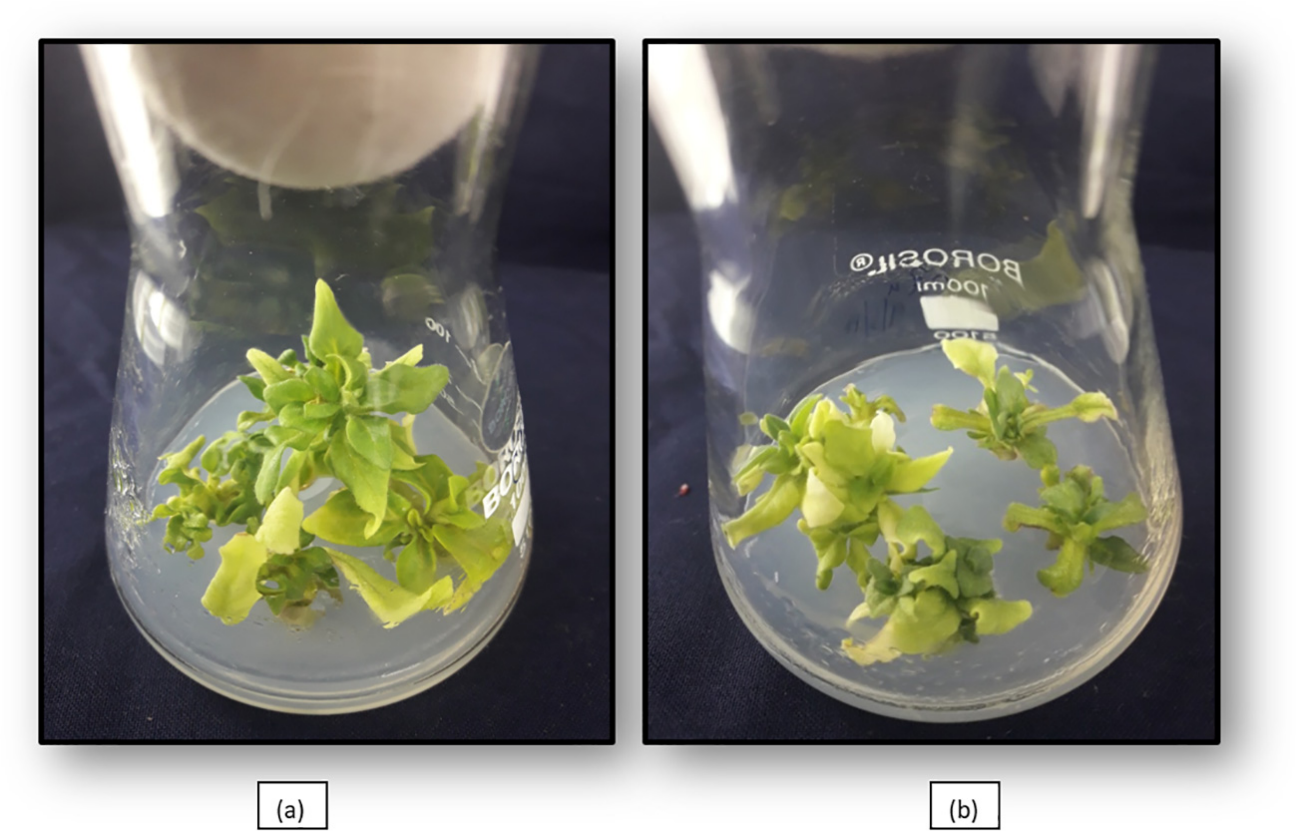
Culture Establishment; (a): IBA + BAP: 0.5 + 1.5 mg L^−1^, (b): IBA + BAP: 0.5 + 1.0 mg L^−1^.

Highest culture asepsis was achieved after 2 weeks of incubation as compared to 4-week duration. The combination of HgCl_2_(0.1%) for 10 min and carbendazim (0.02 and 0.01%) for 30 min followed with 70% ethanol gave the best results in culture sterilization in all the three explants i.e., 89.58%, 89.57% and 83.33% for shoot tip, nodal segment and leaf explants respectively (P ≤ 0.05). However, 10 min HgCl_2_ dip was proved to produce more asceptic cultures than a 5-minute treatment (Table 1). Nodal segment explant observed significantly higher rate of survival (87.49%) than shoot tip (85.41%) and leaf explant (81.33). Significantly higher survival was observed in treatments that involved the 5-minute dip in 0.1% HgCl_2_ as compared to 10 min dip (Table 1). Twelve growth regulator combinations were used for standardization of explant establishment in petunia involving IBA at 0.1, 0.2, 0.3 and 0.5 mg L^−1^ and BAP at 0.5, 1.0 and 1.5 mg L^−1^(Table 2). In shoot tips and nodal segments, highest establishment per cent i.e., 95.82% and 89.57% in shoot tips and nodal segments respectively was observed with IBA at 0.5 mg L^−1^when combined with BAP at 1.5 mg L^−1^, followed by IBA at0.5 mg L^−1^ in combination with BAP at 1.00 mg L^−1^ (Fig. 2).

**Table 1:** Influence of sterilant treatments and time duration on asceptic culture and survival of explants of *Petunia hybrida* Vilm. cv. “Bravo” Figures in the parentheses are the statistically transformed (arc sign and square root) values of percentage data.

Sterilant Treatments		Explant											
		Shoot Tip				Nodal Segment				Leaf			
		Asepsis %		Survival %		Asepsis %		Survival %		Asepsis %		Survival %	
		2 weeks (T_1_)	4 weeks (T_2_)	2 weeks (T_1_)	4 weeks (T_2_)	2 weeks (T_1_)	4 weeks (T_2_)	2 weeks (T_1_)	4 weeks (T_2_)	2 weeks (T_1_)	4 weeks (T_2_)	2 weeks (T_1_)	4 weeks (T_2_)
S1	HgCl_2_ 0.1% for 5 min	45.83 (42.58)	33.33 (35.16)	54.16 (47.37)	41.66 (40.14)	47.91 (6.97)	43.78 (6.66)	54.16 (47.37)	43.74 (41.34)	56.24 (7.55)	45.83 (6.83)	49.99 (44.97)	39.58 (38.91)
S2	HgCl_2_ 0.1% for 10 min	85.41 (67.70)	77.08 (61.45)	47.91 (43.78)	39.58 (38.91)	83.33 (9.12)	77.08 (8.27)	52.08 (46.17)	41.66 (40.14)	81.24 (9.06)	77.08 (8.83)	45.83 (42.58)	35.41 (36.48)
S3	HgCl_2_ 0.1% for 5 min + ethyl alcohol 70% for 10 s	47.91 (43.78)	41.66 (40.14)	79.16 (63.43)	64.58 (53.59)	54.16 (7.42)	45.83 (6.83)	74.99 (60.29)	64.58 (53.55)	62.58 (7.95)	49.99 (7.12)	72.91 (58.81)	62.58 (52.40)
S4	Carbendezim 0.01% for 30 min + S_3_	49.99 (44.98)	41.66 (40.14)	91.66 (75.40)	83.33 (66.22)	60.41 (7.82)	47.91 (6.97)	93.74 (77.23)	85.41 (67.70)	64.58 (8.08)	56.24 (7.56)	89.57 (71.35)	79.16 (62.92)
S5	Carbendezim 0.02% for 30 min + S_3_	54.16 (47.37)	47.91 (43.78)	**93.74 (77.23)**	**85.41 (67.70)**	62.49 (7.96)	50.08 (7.13)	**97.90 (85.34)**	**87.49 (69.53)**	68.74 (8.34)	62.57 (7.96)	**93.74 (79.46)**	**81.33 (64.46)**
S6	Carbendezim 0.01% for 30 min + HgCl_2_ 0.1% for 10 min + ethyl alcohol 70% for 10 s	89.57 (73.62)	85.41 (67.70)	41.66 (40.15)	33.33 (35.16)	87.49 (9.39)	79.16 (8.95)	43.74 (41.34)	37.49 (37.60)	**91.66 (9.62)**	**83.33 (9.18)**	37.49 (37.71)	29.16 (32.61)
S7	Carbendezim 0.02% for 30 min + HgCl_2_ 0.1% for 10 min + ethyl alcohol 70% for 10 s	**93.74 (79.54)**	**89.58 (73.62)**	39.57 (38.94)	31.24 (33.93)	**95.82 (9.83)**	**89.57 (9.51)**	41.91 (40.91)	31.24 (33.85)	91.66 (9.62)	87.49 (9.40)	35.41 (36.48)	29.16 (32.61)
C.D_(P≤0.05)_	Time (T)	**3.39**		**0.24**		**0.21**		**3.02**		**2.93**		**2.67**	
	Sterilant (S)	**6.34**		**0.49**		**0.39**		**5.65**		**5.49**		**5.01**	
	TxS	**NS**		**NS**		**NS**		**NS**		**NS**		**NS**

**Table 2:** Influence of growth regulator combinations on per cent culture establishment in *Petunia hybrida* Vilm. cv. “Bravo”.

Treatments (MS + PGRS)	Shoot tips	Nodal segments
IBA (0.1 mg L^−1^) + BAP (0.5 mg L^−1^)	45.83 (6.83)	31.24 (5.66)
IBA (0.1 mg L^1^) + BAP (1.0 mg L^−1^)	52.08 (7.28)	43.74 (6.66)
IBA (0.1 mg L^−1^) + BAP (1.5 mg L^−1^)	70.83 (8.47)	64.58 (8.08)
IBA (0.2 mg L^−1^) + BAP (0.5 mg L^−1^)	43.74 (6.66)	37.49 (6.19)
IBA (0.2 mg L^−−1^) + BAP (1.0 mg L^−1^)	62.49 (7.96)	58.33 (7.69)
IBA (0.2 mg L^−1^) + BAP (1.5 mg L^−1^)	81.24 (9.06)	72.91 (8.58)
IBA (0.3 mg L^−1^) + BAP (0.5 mg L^−1^)	77.08 (8.83)	77.08 (8.83)
IBA (0.3 mg L^−1^) + BAP (1.0 mg L^−1^)	85.41 (9.29)	79.16 (8.95)
IBA (0.3 mg L^−1^) + BAP (1.5 mg L^−1^)	87.49 (9.40)	83.33 (9.18)
IBA (0.5 mg L^−1^) + BAP (0.5 mg L^−1^)	83.33 (9.17)	79.16 (8.95)
IBA (0.5 mg L^−1^) + BAP (1.0 mg L^−1^)	91.66 (9.62)	87.49 (9.40)
IBA (0.5 mg L^−1^) + BAP (1.5 mg L^−1^)	**95.82 (9.83)**	**89.57 (9.51)**
**C.D_(P_**_≤_**_0.05)_**	**0.47**	**0.55**

Figures in the parentheses are square root transformed values of percentage data.

### 3.2 Callusing, regeneration and organogenesis

**Fig. 3:**
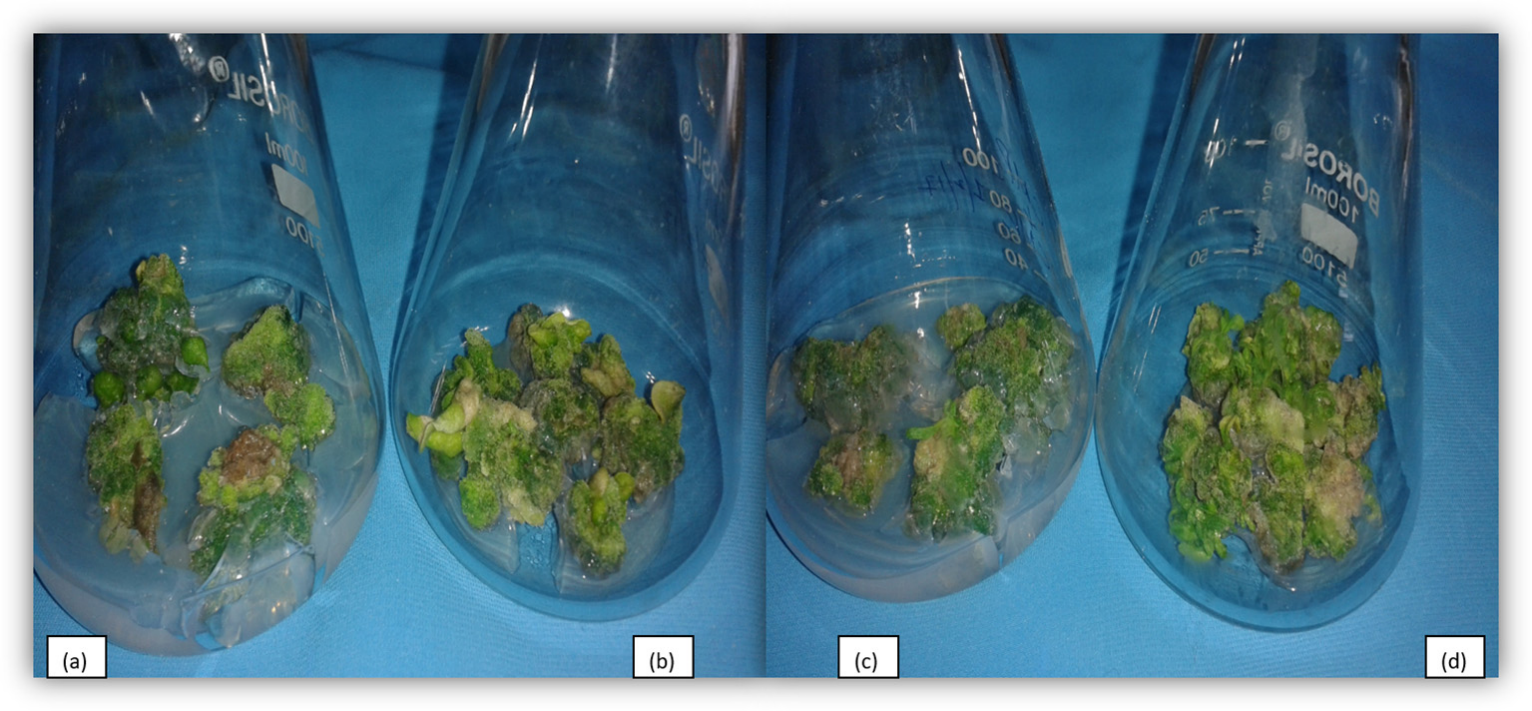
Callusing, (a): Callusing from leaf explants in MS media, BAP + 2,4-D : 1.0 + 1.5 mg L^−1^; (b): Callusing from leaf explants in MS media, BAP + 2,4-D : 1.0 + 1.0 mg L^−1^; (c): Callusing from internodal segment explants in MS media, BAP + 2,4-D : 1.0 + 1.5 mg L^−1^; (d): Callusing from internodal segment explants in MS media, BAP + 2,4-D : 1.0 + 1.0 mg L^−1^.

**Fig. 4:**
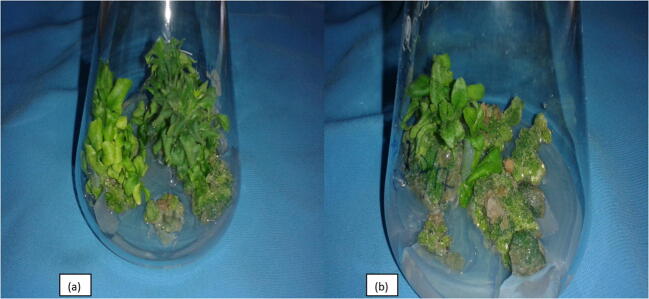
Shoot regeneration, Kinetin + NAA: 2.0 + 0.5 mg L^−1^; (a): Shoot regeneration from leaf derived callus; (b): shoot regeneration from internodal segment derived callus.

Several hormonal combinations were used for callus induction of leaf and internodal segment explants of petunia comprising of auxins (NAA and 2, 4-D each at the concentrations of 0.5, 1.00 and 1.50 mg L^−1^) and cytokinin’s (BAP at the concentration of 0.50 or 1.00 mg L^−1^) (Table 3). Maximum induction of callus (85.41% in leaf explants and 77.08% in internodal segment explants) and maximum weight of the callus (1.78 g explant^−1^ in leaf and 1.82 g explant^−1^) in internodal segment were recorded on MS media containing higher concentrations of 2, 4-D in combination of BAP at 1.50 mg L^−1^ (Fig. 3). NAA (0.50 mg L^−1^) based treatments significantly regenerated high percentage of callus producing shoots and shoot number callus^−1^ than IBA (0.50 mg l^−1^) containing treatment combinations. This may be attributed to more stability of NAA in autoclave than IBA(Table 4). Highest per cent of shoot induction and shoot number per callus piece was recorded on media fortified with Kinetin at 2.00 mg L^1^and NAA 0.50 mg L^1^and then on MS fortified with BAP 2.00 mg L^1^and NAA 0.50 mg L^−1^ in comparison to other treatment combinations (Fig. 4).

**Table 3:** Influence of growth regulators on callusing in leaf and internodal segment explants.

Treatments (MS + PGRS)	Leaf			Internodal Segment		
	*Callus induction (%)	**Callus fresh weight (g explant^−1^)	Callus type	*Callus induction (%)	**Callus fresh weight (g explant^−1^)	Callus type
BAP (0.5 mg L^−1^) + NAA (0.5 mg L^−1^)	77.08 (8.82)	1.39	Compact, yellowish green	60.41 (7.82)	1.47	Loose, creamy green
BAP (0.5 mg L^−1^) + NAA (1.0 mg L^−1^)	72.91 (8.59)	1.28	Compact, yellowish green	54.16 (7.42)	1.31	Loose, creamy green
BAP (0.5 mg L^−1^) + NAA (1.5 mg L^−1^)	56.24 (7.56)	0.90	Compact, green	41.66 (6.51)	1.11	Loose, creamy green
BAP (0.5 mg L^−1^) + 2,4-D (0.5 mg L^−1^)	68.74 (8.34)	0.98	Compact, creamish green	45.83 (6.83)	1.04	Compact, creamy green
BAP (0.5 mg L^−1^) + 2,4-D (1.0 mg L^−1^)	74.99 (8.71)	1.32	Compact, creamish green	64.57 (8.09)	1.36	Compact, creamy green
BAP (0.5 mg L^−1^) + 2,4-D (1.5 mg L^−1^)	79.16 (8.95)	1.44	Loose, creamish green	66.66 (8.21)	1.54	Compact, creamy green
BAP (1.0 mg L^−1^) + NAA (0.5 mg L^−1^)	83.33 (9.17)	1.58	Compact, yellowish green	72.91 (8.59)	1.75	Compact, green
BAP (1.0 mg L^−1^) + NAA (1.0 mg L^−1^)	85.41 (9.29)	1.78	Compact, green	77.08 (8.83)	1.82	Compact, green
BAP (1.0 mg L^−1^) + NAA (1.5 mg L^−1^)	81.24 (9.06)	1.50	Compact, green	68.74 (8.34)	1.67	Compact, green
BAP (1.0 mg L^−1^) + 2,4-D (0.5 mg L^−1^)	83.33 (9.17)	1.56	Compact, creamish green	74.99 (8.71)	1.73	Compact, creamy green
BAP (1.0 mg L^−1^) + 2,4-D (1.0 mg L^−1^)	91.66 (9.62)	1.88	Compact, brownish green	87.49 (9.40)	2.05	Compact, creamy green
BAP (1.0 mg L^−1^) + 2,4-D (1.5 mg L^−1^)	95.82 (9.83)	1.90	Loose, brownish green	91.66 (9.62)	2.11	Compact, green
**C.D_(P_**_≤_**_0.05)_**	**0.48**	**0.16**		**0.54**	**0.16**	

Figures in the parentheses are square root transformed value of the percentage data.

* Data recorded after 4 weeks of culture.

** Callus weight recorded after 6 weeks of culture.

**Table 4:** Influence of growth regulator combinations on shoot regeneration in leaf and internodal segment derived callus.

Treatments (MS + PGRS)	Leaf Derived Callus		Internodal Segment Derived Callus	
	Regeneration (%)	Shoot number callus^−1^	Regeneration (%)	Shoot number callus^−1^
BAP (1.0 mg L^−1^) + IBA (0.5 mg L^−1^)	4.16 (2.02)	1.25	2.08 (1.51)	1.75
BAP (1.0 mg L^−1^) + NAA (0.5 mg L^−1^)	18.74 (4.33)	7.50	16.66 (4.14)	5.50
Kinetin (1.0 mg L^−1^) + IBA (0.5 mg L^−1^)	6.24 (2.54)	2.50	4.16 (2.02)	2.50
Kinetin (1.0 mg L^−1^) + NAA (0.5 mg L^−1^)	22.91 (4.84)	9.00	20.83 (4.65)	8.75
BAP (2.0 mg L^−1^) + IBA (0.5 mg L^−1^)	8.33 (2.82)	5.75	8.33 (2.82)	4.00
BAP (2.0 mg L^−1^) + NAA (0.5 mg L^−1^)	27.08 (5.25)	10.50	27.08 (5.25)	12.00
Kinetin (2.0 mg L^−1^) + IBA (0.5 mg L^−1^)	14.58 (3.85)	6.00	12.49 (3.62)	6.25
Kinetin (2.0 mg L^−1^) + NAA (0.5 mg L^−1^)	35.41 (6.02)	13.00	29.16 (5.47)	14.50
**C.D_(P_**_≤_**_0.05)_**	**1.46**	**2.36**	**1.31**	**2.63**

Data recorded after 8 weeks of culture.

Figures in the parentheses are square root transformed value of the percentage data.

### 3.3 Shoot proliferation

**Fig. 5:**
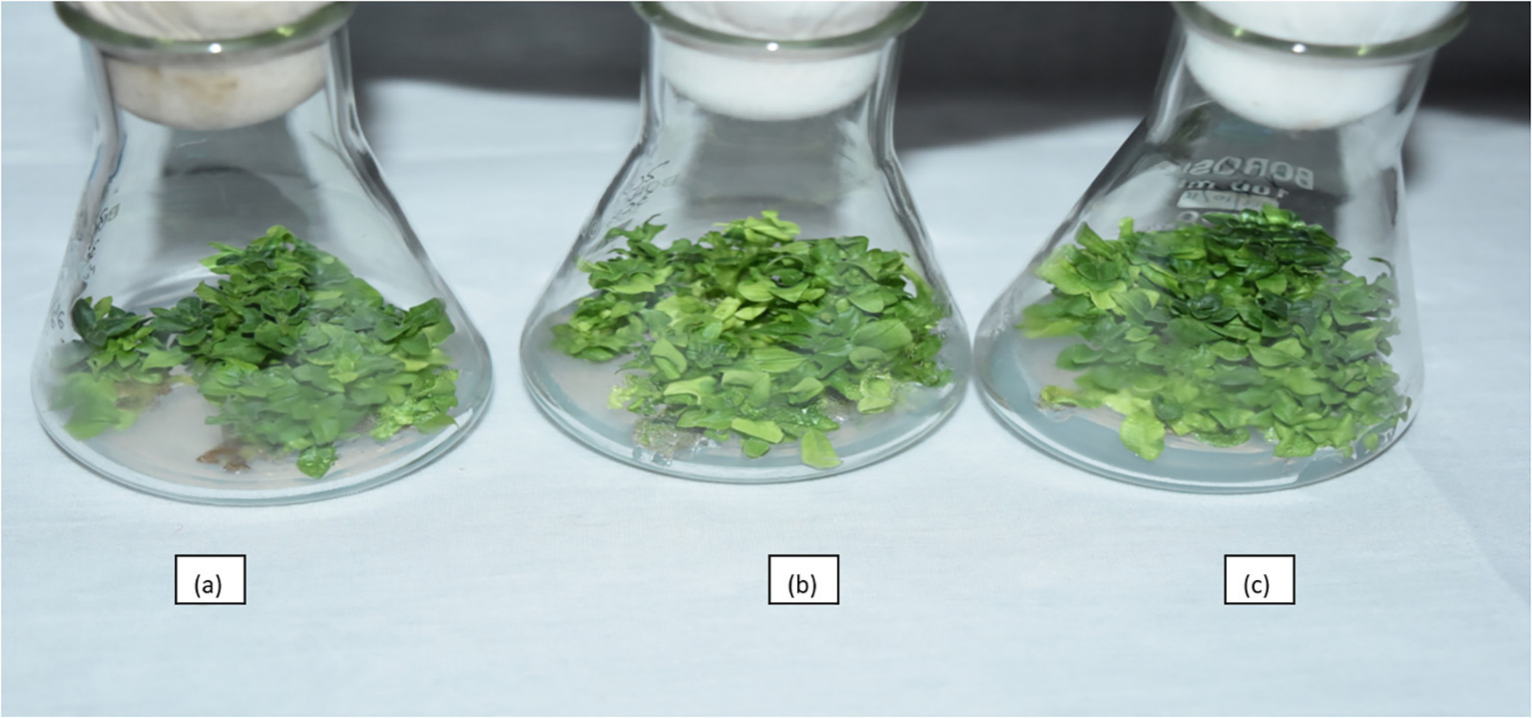
Shoot proliferation of microshoots of *Petunia hybrida*; (a): BAP (1.00 mg L^−1^) + IBA (0.50 mg L^−1^); (b): BAP (0.50 mg L^−1^) + IBA (0.25 mg L^−1^); (c): BAP (0.50 mg L^−1^) + IBA (0.50 mg L^−1^).

Initial shoots raised *in vitro* from healthy pre-established nodal segments and shoot tips were employed for the purpose. Six growth regulator treatment combinations involving BAP at 0.50 and 1.00 mg L^−1^ with IBA 0.10, 0.25 and 0.50 mg L^−1^ were used in this experiment. Significantly high per cent shoot proliferation (97.90%), shoot number (22.25 explant^−1^) and maximum shoot length (2.70 cm) was achieved at PGR combination of BAP 0.5 + IBA 0.50 mg L^−1^, followed by same concentration of BAP combined with IBA 0.25 mg L^−1^ (Table 5), (Fig. 5).

**Table 5:** Influence of growth regulator combinations on shoot proliferation from microshoots of *Petunia hybrida* Vilm cv. “Bravo”.

Treatments (MS + PGRS)	Shoot proliferation (%)	*Shoot number explant^−1^	*Shoot length
BAP (0.50 mg L^−1^) + IBA (0.10 mg L^−1^)	70.83 (8.47)	8.25	1.52
BAP (0.50 mg L^−1^) + IBA (0.25 mg L^−1^)	91.66 (9.62)	21.50	2.53
BAP (0.50 mg L^−1^) + IBA (0.50 mg L^−1^)	97.90 (9.94)	22.25	2.70
BAP (1.00 mg L^−1^) + IBA (0.10 mg L^−1^)	72.91 (8.59)	9.50	1.95
BAP (1.00 mg L^−1^) + IBA (0.25 mg L^−1^)	77.08 (8.82)	18.00	1.87
BAP (1.00 mg L^−1^) + IBA (0.50 mg L^−1^)	81.24 (9.06)	17.50	2.47
**C.D_(P_**_≤_**_0.05)_**	**0.46**	**3.04**	**0.39**

Figures in the parentheses are square root transformed values of percentage data.

* Data recorded after 6 weeks of culture.

### 3.4 Rhizogenesis and hardening

**Fig. 6:**
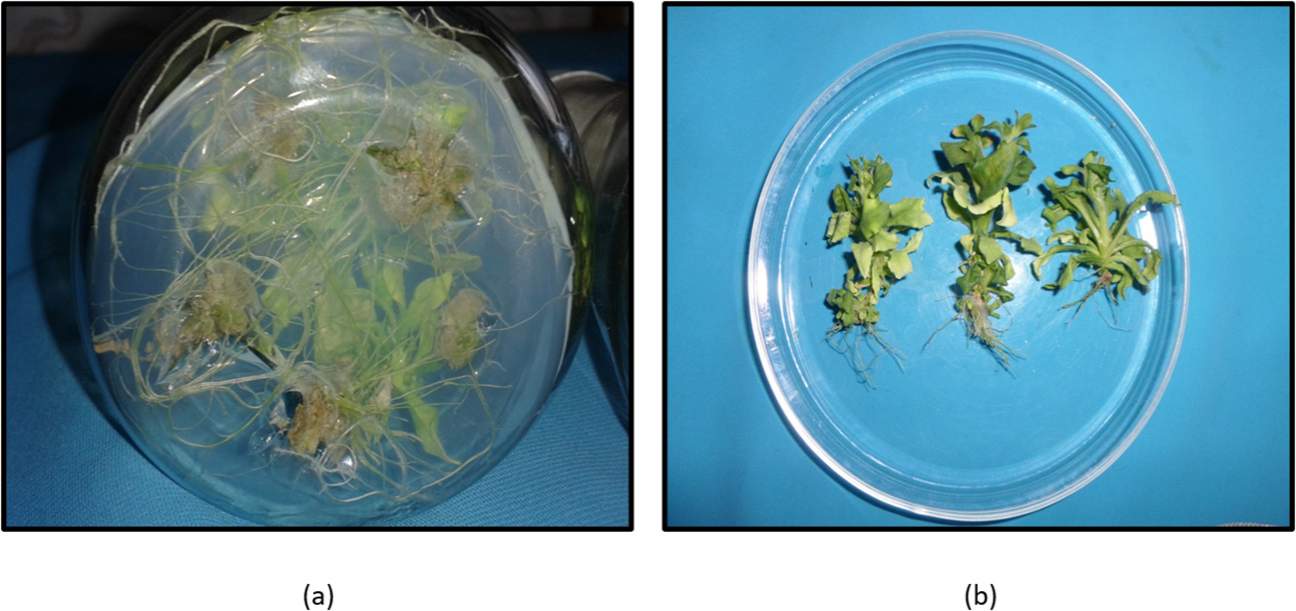
Rhizogenesis of *Petunia hybrid;* (a): IBA 1.00 mg L^−1^; (b): IBA 0.75 mg L^−1^; NAA 1.00 mg L^−1^.

**Fig. 7:**
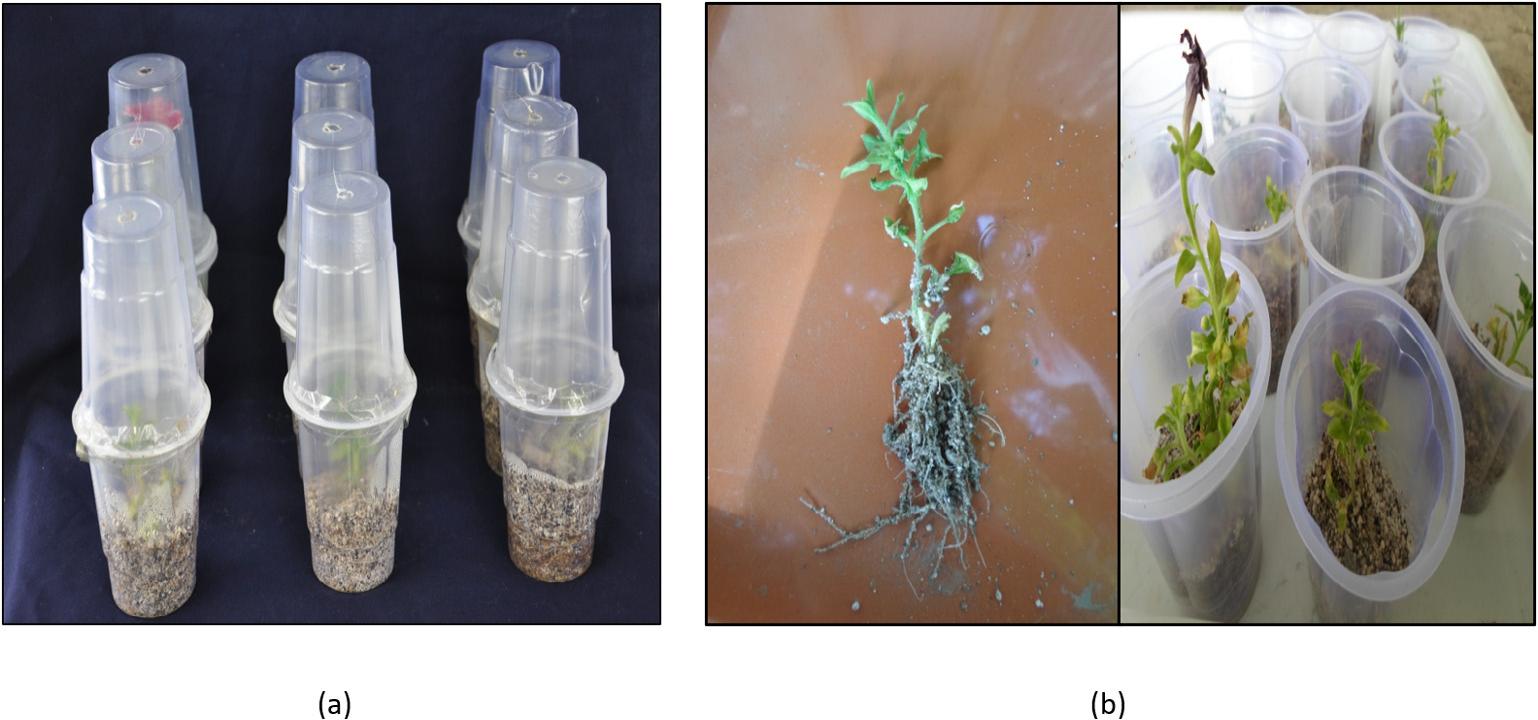
(a): Hardening Stage; (b): Rooted plantlets during hardening.

The rooting characteristics (per cent rooting and root number shoot^−1^) of *Petunia hybrida* Vilm cv. “Bravo” improved significantly as IBA and NAA concentration was elevated from 0.25 to 1.00 mg L^−1^. However IBA was recorded to have more effect than NAA (Table 6). Significantly highest rooting per cent i.e., 95.82% and root number shoot^−1^ i.e., 8.00 was recorded on media having 1.00 mg L^−1^ IBA followed by IBA 0.75 mg L^−1^ (Fig. 6). Plantlet survival was observed highest (92.50%) in media HM_6_ (perlite + vermiculite: 1:1) followed by HM_5_ (vermiculite) and HM_4_ (perlite) with 86.45 and 83.75 per cent, respectively (Table 7). Hardened plantlets have been shown in Fig. 7.

**Table 6:** Influence of auxins on Rhizogenesis in *Petunia hybrida.*Vilm cv. “Bravo”.

Treatments (MS + PGRS)	Rooting (%)	Root number shoot^−1^
IBA (0.25 mg L^−1^)	35.41 (36.40)	2.25
IBA (0.50 mg L^−1^)	52.08 (46.17)	4.75
IBA (0.75 mg L^−1^)	89.57 (71.35)	7.75
IBA (1.00 mg L^−1^)	95.82 (81.28)	8.00
NAA (0.25 mg L^−1^)	31.24 (33.85)	1.50
NAA (0.50 mg L^−1^)	41.66 (40.14)	3.50
NAA (0.75 mg L^−1^)	62.49 (52.36)	5.00
NAA (1.00 mg L^−1^)	74.99 (60.13)	6.25
**C.D_(P_**_≤_**_0.05)_**	**8.17**	**1.43**

Figures in the parenthesis are arcsine transformed values of percentage data.

*Data recorded after 4 weeks of growth.

**Table 7:** Influence of hardening media on *ex vitro* survival of rooted plantlets.

Hardening Media	Survival (%)
HM1 : Sand	36.25 (36.93)
HM2 : Sand + Perlite (1:1)	60.00 (50.76)
HM3 : Sand + Vermiculite (1:1)	62.50 (52.5)
HM4 : Perlite	83.75 (66.38)
HM5 : Vermiculite	86.25 (68.41)
HM6 : Perlite + Vermiculite (1:1)	92.50 (74.29)
C.D_(P≤0.05)_	**5.14**

Figures in the parenthesis are arcsine transformed values of percentage data.

## 4 Discussions

### 4.1 Culture asepsis and explant establishment

Choosing the correct explant is essential if the desired outcome of any tissue culture procedure is to be achieved with minimal delays, besides proper sterilization of the explants is the pre-requisite step leading to the development of a successful protocol for *in vitro* propagation. More asepsis was observed in 2 week cultures than the 4 week cultures. This may be attributed to the endophytic pathogens that come out and cause infection after 4 weeks thus increasing the overall contamination. After washing explants with tap water, several workers have used different sterilants like mercuric chloride, sodium hypochloride or ethyl alcohol for the disinfection singly or in combination with fungicide as bavistin (a.i. carbendazim). Mercuric chloride being a potent and efficient surface sterilant has been widely and extensively used in petunia explants by most of the researchers. Mishra et al. (2006) reported the use0.1% HgCl_2_for 8 min as sterilization treatment for shoot tip, leaf segments, internodal segments and nodal stems, and leaf segments of petunia. Similarly, Li et al., 2013, Natalija et al., 2015 also had satisfactorily surface sterilized petunia explants by using 70% ethanol for 10–30 s. The combination of carbendazim and mercuric chloride has been used effectively by many researchers to achieve a required measure of *in-vitro* culture asepsis. (Thakur et al., 2002, Beura et al., 2005). Also, the concentration and duration of the sterilant treatments have not only proven to be the factors of utmost importance in the process of explant sterilization but also to ensure the explant survival with minimum toxicity. Nodal segments survived in a significant way when compared to leaf and internodal segments as nodal sections are highly compacted with toughened surface fibres (sclerenchymatous tissues) that are able to tolerate the effects of to mercuric chloride. Furthermore, shoot tip survival was reported to be higher than leaf which may be due to their secure location due to outer leaf tiers that are separated after sterilization. Sterilant treatments were proved to be having toxic effects on leaf explants which may be due to its thin epidermis, thus providing less protection against the lethal effect of sterilants. Many researchers have achieved maximum survival in different flower explants of of several plants (Petunia, Carnation and Lily) when exposed to HgCl_2_ (0.1%) for shorter duration in sterilization process. (Mishra et al., 2006, Sooch et al., 2000, Dilta et al., 2000).

The important part played by BAP as an important hormone for shoot differentiation and proliferation in petunia was confirmed by Economou and Read (1981). The significant effect of high cytokinin: auxin ratio for multiple shoot induction has been reported in the same family *Solanaceae* (Khadiga et al., 2009). Clapa and Cantor (2006) reported that *in vitro* shoot regeneration of petunia occurred on MS media fortified with 1 mg L^−1^ BA. Sabitha et al. (2009) noticed that multiple number of shoots formed in stem explants of *Petunia hybrida* on MS media containing BAP at the concentration of 3 mg L^−1^.

### 4.2 Callusing, regeneration and organogenesis

Auxins play a key role in cell division as well as in increasing cell volume by loosening up the cell walls thus allowing more water uptake hence increasing the weight of the cell. The inferences obtained in our study are in conformity with Huang et al. (2002), who reported higher values for callus induction in leaf discs of *P. hybrida* in medium supplemented with 2,4-D at 0.5 mg L^−1^ or BA at 0.5 mg L^−1^ + 2,4-D at 0.5 mg L^−1^. Kanwar and Kumar (2009) have also reported maximum callus development in internodes of carnation in 2, 4-D at 2.00 mg L^−1^and BA at 1.00 mg L^−1^with minimum callus development on media having Kinetin and NAA. Thenmozhi and Sivaraj (2011) also observed significantly higher percentage of callus induction in 2,4-D at 1.5 mg L^−1^ followed by 2.0 and 1.0 and 0.5 mg L^−1^for leaf explants of Petunia. Similarly Sherkar and Chavan (2014) noted that 2, 4-D at the concentration of 3.0 mg L^−1^ was recorded to be the most effective for induction of callus in potato explants that belongs to the same family. Also, MS media with 2, 4-Drecorded higher callus weight when compared to NAA fortified MS media. Vidya et al., 2013, Mahadev et al., 2014 have demonstrated that auxin and cytokinin combination supports organogenesis in the callus. The results obtained were in conformity with many workers who reported shoot regeneration from callus derived from leaf explant of petunia on MS containing NAA and BAP. (Curry, 2000, Seema et al., 2003, Wu and Li, 2007, Shrin et al., 2007, Kumar et al., 2014).

### 4.3 Shoot proliferation

Shoot proliferation from axillary branching method is to be known as most widely successful course for *in vitro* mass propagation of petunia. Cytokinin’s at appropriate concentration levels enhance cell division and inhibit apical dominance, thereby help in promotion of shoot proliferation from axillary buds. Therefore, for the mass multiplication of any plant species, a suitable cytokinin concentration level is a pre-requisite for optimum shoot production. GhaffariEsizad et al. (2012) have reported positive effect of cytokinin on multiplication and proliferation rate of Lisianthus. A number of workers have tried various PGRs either alone or in combinations with each other for shoot proliferation with diverse results in petunia. Cui et al. (2005) recorded the effects of combination of various concentrations of cytokinin with 0.10 mg NAA l^−1^ on shoot propagation and proliferation of *Petunia hybrida* and found the suitable media for shoot propagation as MS + 1.60 mg L^−1^ BA + 0.10 mg L^−1^ NAA. Mishra *et. al*. (2006) supplemented MS media with 1.0 mg BAP L^−1^ and 0.1 mg L^−1^of IBA which resulted in significantly highest shoots explants^−1^ as well as elongation of shoots in *Petunia hybrida*. Mohamed (2011) has observed shoot proliferation in carnation cv. ‘White Sim’ from nodal explants on MS media containing 8.87 µM BAP.

### 4.4 Rhizogenesis and hardening

Auxins, especially IBA are known to significantly improve the rooting per cent and its quality. Most of the workers achieved stimulated rooting of petunia on media fortified with auxins as auxins have a good potential to promote root initiation (Wetherell, 1982). Qu and Qu (2001) obtained rooting on stem segments of *Petunia hybrida* using the treatment combination MS + NAA 0.5 mg L^−1^ and MS + NAA 0.3 mg L^−1^with IBA 0.2 mg L^−1^. Cui et al. (2005) also determined the effects of auxins on rooting of shoots in *Petunia hybrida* by using 0.20 mg L^−1^IBA, and 0.20 mg L^−1^NAA. Mishra et al.(2006) observed rooting in Petunia cv. “Cascade Burgundy” in half-basal MS media that contained auxin combination of 0.1 mg L^−1^IBA + 0.1 mg L^−1^NAA. Sabitha et al. (2009) also achieved IBA at 20 µM dissolved in MS media was found to be better for rooting. Atak and Celik (2009) transferred the regenerated shoots of *Anthurium adreanum* cv. “Arizona” to the medium supplemented with 1 mg l^−1^ IBA which produced good quality rooting. In another study, IBA was observed to be more successful in root induction of capsicum plants than NAA even alone or in combination with each other (Otroshy et al. 2011). Plantlets developed *in-vitro* need to be acclimatized for some weeks in low humid conditions before finally transferring them to the field conditions (Bolar et al., 1998). Thus, hardening media has a key importance in *ex vitro* establishment of the plants developed *in vitro*. The use of vermiculite as an effective hardening media for *in vitro* rooted plantlets was reported by many researchers. LiNa et al. (2006) has also observed 90 per cent survival rate when plantlets of carnation were transplanted to the substratum consisting of equal proportions of perlite and vermiculite. All acclimated plants were then transferred to the open conditions which grew normally in the natural environment.

## 5 Conclusion

For the development of propagation protocol for *Petunia hybrida* cv. “Bravo”, various steps were followed. Maximum uncontaminated growing cultures of petunia were obtained with 0.02% Carbendezim for 30 min followed by HgCl_2_ at 0.1% for the duration of 10 min with a final treatment of10 second wash with 70% ethanol.The highest percentage of culture establishment was observed in MS- liquid media that contained the combination of plant growth regulator IBA 0.5 mg L^−1^ + BAP 1.5 mg L^−1^. Maximum induction of callus was achieved with BAP at 1.0 mg L^−1^ and 2,4-D at 1.5 mg L^−1^.Highest regeneration from callus was obtained onMS media fortified with Kinetin 2.0 mg L^−1^ and IBA 0.5 mg L^−1^. Proliferation of petunia hybrida was highest in media containing 0.50 mg L^−1^of BAP and 0.50 mg L^−1^ of IBA. Best rooting was observed in MS media containing1.00 mg L^−1^IBA. Highest hardening survival was achieved media that contained equal ratio of perlite and vermiculite mix.

## 6 Ethics approval

Not applicable.

## 7 Consent to participate

All authors consent to participate in this manuscript.

## 8 Consent for publication

All authors consent to publish this manuscript in Saudi Journal of Biological Science.

## 9 Availability of data and material

Data will be available on request to corresponding or first author.

## 10 Code availability

Not applicable.

## Author contributions

IF, ZAQ, and SM drafted the experimental design. IF, ZAR, ITN, and NB performed the experiments. AN, HD, SR, KZM, SSA and SM helped in data collection, data analysis and initial draft of manuscript text. All authors read the manuscript before communication.

## Declaration of Competing Interest

The authors declare that they have no known competing financial interests or personal relationships that could have appeared to influence the work reported in this paper.
